# A hybrid deep learning framework for air quality prediction with spatial autocorrelation during the COVID-19 pandemic

**DOI:** 10.1038/s41598-023-28287-8

**Published:** 2023-01-18

**Authors:** Zixi Zhao, Jinran Wu, Fengjing Cai, Shaotong Zhang, You-Gan Wang

**Affiliations:** 1 College of Mathematics and Physics, Wenzhou University, Wenzhou, 325035 People’s Republic of China; 2 The Institute for Learning Sciences and Teacher Education, Australian Catholic University, Brisbane, 4000 Australia; 3 Frontiers Science Center for Deep Ocean Multispheres and Earth System, Key Lab of Submarine Geosciences and Prospecting Techniques, MOE and College of Marine Geosciences, Ocean University of China, Qingdao, 266100 People’s Republic of China

**Keywords:** Engineering, Mathematics and computing

## Abstract

China implemented a strict lockdown policy to prevent the spread of COVID-19 in the worst-affected regions, including Wuhan and Shanghai. This study aims to investigate impact of these lockdowns on air quality index (AQI) using a deep learning framework. In addition to historical pollutant concentrations and meteorological factors, we incorporate social and spatio-temporal influences in the framework. In particular, spatial autocorrelation (SAC), which combines temporal autocorrelation with spatial correlation, is adopted to reflect the influence of neighbouring cities and historical data. Our deep learning analysis obtained the estimates of the lockdown effects as − 25.88 in Wuhan and − 20.47 in Shanghai. The corresponding prediction errors are reduced by about 47% for Wuhan and by 67% for Shanghai, which enables much more reliable AQI forecasts for both cities.

## Introduction

Air pollution has long been a major matter of concern in China^1^. Exposure to harmful air pollution for a long time will result in a range of respiratory ailments, cardiovascular diseases, and even lung cancer in humans^2^. Furthermore, high concentrations of air pollutants harm food production and imperil animal survival^3^. Hence, rational prediction of air quality provides a level of protection for humans and nature.

As a typical time series, air quality is affected by not only seasonal factors but also by significant social factors^4^. For example, at the end of 2019, a new coronavirus broke out in Wuhan, China, which was easily transmitted through the air. To cut off the transmission of the virus, the Wuhan government implemented a 76-day lockdown policy limiting human activities, which in turn positively improved air quality^5,6^, because the concentrations of $\text {PM10}$, $\text {PM2.5}$, $\text {NO}_2$ and CO from vehicle exhaust and industry decreased dramatically^7^. According to Lian et al.^8^, the $\text {NO}_{2}$ concentration and AQI decreased by 53.2% and 33.9%, respectively, during the lockdown period in Wuhan. To some extent, the improvement of air quality during the epidemic is an opportunity to spark new pollution management ideas from the government, such as the scheduling of traffic and industrial production. Therefore, accurate air quality prediction during the epidemic is of social importance.

### Literature review

Air quality prediction is a hot topic in the environmental field, and the common prediction methods are three main categories: numerical simulation, statistical methods, and machine learning. Earlier studies on air quality prediction mostly used numerical simulation. Using mathematical knowledge, it builds models to simulate changes in air quality based on chemical and physical processes in the atmosphere. The classical models are the nested air quality prediction modelling system^9^, weather research and forecasting model^10,11^, and community multiscale air quality model^12,13^. However, these models place high demands on the dataset and assume that the pollution discharge is constant, which is not true since pollutants are emitted randomly in fact^14^. Besides, numerical simulation methods often produce complex calculations, which are not user-friendly. In view of these inadequacies, statistical methods to predict air quality have become increasingly popular among researchers.

The statistical method does not involve meteorological theories; instead, it mainly explores patterns from the data to construct prediction models^15–17^. Considering that air quality data is a typical time series, auto regressive moving average model (ARMA) is widely used. Kumar et al.^18^ used ARMA to predict $\text {O}_{3}$, CO, NO and $\text {NO}_{2}$ concentrations, and the model achieved good performance at an urban traffic site in Delhi, India. Regression models are also well suited to address prediction problems. Stadlober et al.^19^ constructed a multiple linear regression model that combined current data with the next day meteorological forecasts to predict the daily $\text {PM10}$ concentrations, which assisted the government in making traffic control decisions. However, most statistical methods require the independent and dependent variables to be linearly correlated, while there is significant nonlinearity between air quality data^20^. Therefore, statistical methods sometimes do not achieve satisfying results.

Machine learning has been a popular choice for air quality forecasting because it is good at dealing with nonlinear problems. Dai et al.^21^ set up a hybrid model by using a multilayer perception that could predict the $\text {PM2.5}$ concentration and fluctuation in different regions more effectively. Ketu et al.^22^ combined the adjustment of kernel scales with a support vector machine^23^, which allows for an accurate classification of air quality. Lim et al.^24^ combined multiple machine learning algorithms to construct a land use regression model for $\text {PM2.5}$ concentration prediction in Seoul, Korea, and experimentally demonstrated that machine learning can further improve model performance. Ma et al.^25^ used a nonlinear extreme gradient boosting to predict air quality in the U.S. which also measured the importance of the variables. Although machine learning algorithms have usually performed well, they still have limitations in terms of their capacity to make multistep predictions and collect long-term data properties.

Deep learning is a branch of machine learning^26^. Among the many algorithms for deep learning, the long short-term memory network (LSTM) is often used to predict air quality due to its effectiveness in solving long-distance dependence^27–29^. For example, Li et al.^30^ used LSTM to predict hourly $\text {PM2.5}$ concentrations in Beijing, and the experimental results proved that the model outperformed ARMA and support vector regression. Cheng et al.^31^ used a variant of LSTM, the bidirectional LSTM (Bi-LSTM), for air quality prediction at stations with missing data, and the strategy reduced the root mean square error by 35.21% on average. Therefore, given the above, it is viable to adopt deep learning models for air quality studies.

Feature selection is often used in combination with deep learning to improve algorithm efficiency. Metaheuristic algorithms are widely used for their simplicity, flexibility, and ability to avoid local optima^32^. Typical representative methods are the genetic algorithm^33,34^, the ant colony optimization^35^, and the particle swarm optimization^36,37^. Later on, a reinforcement learning based bee swarm optimization (QBSO) is proposed for feature selection to obtain a more intelligent optimizer^38^. The QBSO algorithm in feature selection takes the advantage of reinforcement learning with very adaptive and efficient each process, and the QBSO has been popularly used in practice^17,39^.

Although deep learning combined with feature selection has an expectation of improving the prediction accuracy of air quality, it is not yet possible to analyse the spatial characteristics of air quality data. Currently, many scholars are beginning to notice this important characteristic of air quality data, and the spatial correlations have been shown to improve prediction accuracy in many research^17,28,40,41^. In particular, the statistical-based method for spatial correlation modelling is popularly used due to its solid foundation. For example, Huang et al.^42^ predicted $\text {PM2.5}$ concentrations in Beijing by using dynamic spatial correlations among monitoring stations, and the results show that the mean square error of the proposed model is reduced by 15%. Wen et al.^43^ incorporated historical air pollutant concentrations at the target site and neighbouring sites into the model and combined convolutional neural networks and LSTM to extract high-level spatial features. In addition, the graph network also is a great alternative for modelling spatial correlation, and some interesting work can be found in Qi et al.^44^, Gao et al.^45^, and Zhou et al.^46^. Here, it shall be noted that our work focuses on spatial correlation modelling with statistical-based methods.

### The motivation

A summation of the above-mentioned literature reveals the following problems with the previous studies in terms of air quality prediction: (1) The lockdown policy during the COVID-19 pandemic led to sudden changes in air quality, and not considering this factor may produce inadequate predictions. (2) When using metaheuristic feature selection methods to improve model efficiency, high feature dimensionality tends to incur high computational costs. (3) Ignoring the spatiotemporal characteristics of air quality may violate the assumptions of some models, such as a requirement for variable independence, which may reduce the prediction accuracy.

### The contribution

To address the shortcomings of previous works, the goal of this study is to develop a multistep predictive framework based on spatiotemporal effects using deep learning. The following are the main contributions of this study:

- In our work, not only pollutants and meteorological factors, but also social factors (e.g., the lockdown policy during COVID-19) are considered dependent variables for predicting AQI. Multiple linear regression is used to remove the effects of seasonal and epidemic factors on the original series to facilitate the analysis of the potential information of the series.
- A hybrid metaheuristic feature selection method is used to eliminate low correlated variables and reduce the computational cost of the model while avoiding overfitting due to many variables.
- A time-series regression model is used to obtain the residual series, and combining the spatial dependence structure, we construct the spatial autocorrelation variable. Then, using *K*-nearest neighbour mutual information, the spatial autocorrelation variable with the strongest dependence is selected, which can reflect the spatiotemporal characteristics of the AQI.
- LSTM and Bi-LSTM are used to achieve multistep prediction of AQI and compare them with several benchmarks including feedforward neural networks and recurrent neural networks. Through multiple sets of experiments, this paper verifies that the proposed framework can accurately monitor air quality changes.

## The preliminaries

### *K*-nearest neighbour mutual information

In probability and information theory, the mutual information (MI) is a measure of the interdependence between the variables^47^. The common MI formula is for discrete variables. When the measured variables are continuous, their MI needs to be estimated by the *K*-nearest neighbour (KNN), which is the *K*-nearest neighbour mutual information (KNN-MI). Unlike the correlation coefficient, the KNN-MI is not limited by sample size and is more suitable for time series^48^. Suppose we want to obtain the MI between the continuous variables *X* and *Y*. The point pair consisting of (*X*, *Y*) is denoted as *W*. The maximum Euclidean distance between the samples is used as the criterion for selecting the nearest neighbour^49^:

1 $$\begin{aligned} \left\| w_i-w_j\right\| =\max \left\{ \left\| x_i-x_j\right\| ,\left\| y_i-y_j\right\| \right\} . \end{aligned}$$

The distance from $w_i$ to its *k*-th neighbour is denoted as $\frac{1}{2}\delta (i)$. The projection of this distance to the *X*-direction and *Y*-direction is denoted as $\frac{1}{2}\delta _{x}(i)$ and $\frac{1}{2}\delta _{y}(i)$, respectively. Obviously, $\delta (i)=\max \left\{ \delta _{x}(i), \delta _{y}(i)\right\}$.

**Figure 1:**
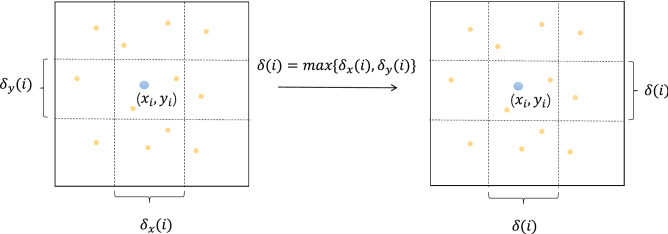
An example of KNN-MI calculation.

Count the number of samples whose distance to $x_i$ is less than $\frac{1}{2}\delta (i)$, denoted as $n_{x}(i)$; and similarly for *y*. Taking Fig. 1 as an example, when $k=1$, $n_{x}(i) = 6$ (horizontally) and $n_{y}(i) = 4$ (vertically). The estimation for MI is as follows:

2 $$\begin{aligned} I(x ; y) \approx \psi (k)-<\psi \left( n_{x}+1\right) +\psi \left( n_{y}+1\right) >+\psi (N), \end{aligned}$$

where $\langle \cdot \rangle$ denotes the mean value; $\psi (x)$ is the digamma function, and $\psi (x)=\frac{d \ln {(\Gamma (x))}}{d x}$. It follows that $\psi (x+1)=\psi (x)+\frac{1}{x}$ and $\psi (1)=$ $-C$, where $C=0.5772156$ is the Euler-Mascheroni constant.

## Proposed AQI forecasting model

### Overall framework

**Figure 2:**
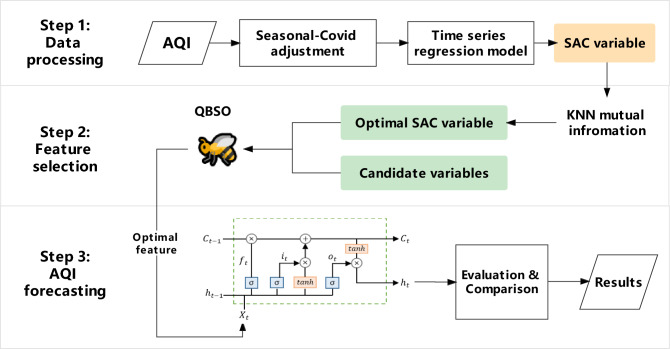
The overall of the proposed AQI forecasting framework.

An overview of the proposed model for AQI prediction is shown in Fig. 2. Besides, this section provides a comprehensive description of the modelling procedure.

### Lockdown adjustment

The purpose of seasonal adjustment, which is the estimation and removal of seasonal effects from a time series, is to uncover the underlying trends of a monthly or quarterly series^50^. When a special event occurs in the selected period, we also need to exclude the effect of that event to analyse the basic characteristics of the original series. Therefore, in this paper, we use a lockdown adjustment to disentangle the original series. The adjusted actual values will be decomposed into three parts, systematic seasonal effects, short-term COVID effects and irregular fluctuations. Using the adjusted values for forecasting allows for the exclusion of differences arising from seasonality and the COVID-19 lockdown policy. We develop an additive time series model with variables containing seasonal terms, epidemic terms, and their interaction terms, as follows:

3 $$\begin{aligned} \begin{array}{rl} S_t =&{} a_0 + a_1 t + a_2 \text {sin\_Yearly} + a_3 \text {cos\_Yearly} + a_4 \text {sin\_Seasonly} + a_5 \text {cos\_Seasonly} \\ &{} + a_6 \text {sin\_Monthly} + a_7 \text {cos\_Monthly} + a_8 \text {sin\_Weekly} + a_9 \text {cos\_Weekly}\\ {} &{} + a_{10} \text {Lockdown} + a_{11} \text {sin\_Yearly\_Lockdown}+ a_{12} \text {cos\_Yearly\_Lockdown}\\ {} &{} + a_{13} \text {sin\_Seasonly\_Lockdown} + a_{14} \text {cos\_Seasonly\_Lockdown}\\ {} &{}+ a_{15} \text {sin\_Monthly\_Lockdown}+ a_{16} \text {cos\_Monthly\_Lockdown} \\ {} &{} + a_{17} \text {sin\_Weekly\_Lockdown} + a_{18} \text {cos\_Weekly\_Lockdown}, \end{array} \end{aligned}$$

where $a_0$ is the intercept; $a_1,\ldots ,a_{18}$ are the coefficients of the equation; and *t* is the observation time. The meaning of each variable is shown in Table 1.

**Table 1:** The meaning of variables used in the COVID adjustment.

Variable		Variable	
*t*	= $1,2,\cdots$	Lockdown	= 1 (in lockdown) or 0
sin_Yearly	= $sin(\frac{2 \pi t}{T_y}),T_y =365.25$	sin_Yearly_Lockdown	sin_Yearly $\times$ Lockdown
cos_Yearly	= $cos(\frac{2 \pi t}{T_y}),T_y=365.25$	cos_Yearly_Lockdown	cos_Yearly $\times$ Lockdown
sin_Seasonly	= $sin(\frac{2 \pi t}{T_s})$,$T_s=\frac{365.25}{4}$	sin_Seasonly_Lockdown	sin_Seasonly $\times$ Lockdown
cos_Seasonly	= $cos(\frac{2 \pi t}{T_s})$,$T_s=\frac{365.25}{4}$	cos_Seasonly_Lockdown	sin_Seasonly $\times$ Lockdown
sin_Monthly	= $sin(\frac{2 \pi t}{T_m})$,$T_m=\frac{365.25}{12}$	sin_Monthly_Lockdown	sin_Monthly $\times$ Lockdown
cos_Monthly	= $cos(\frac{2 \pi t}{T_m})$,$T_m=\frac{365.25}{12}$	cos_Monthly_Lockdown	sin_Monthly $\times$ Lockdown
sin_Weekly	= $sin(\frac{2 \pi t}{T_w})$,$T_w=7$	sin_Weekly_Lockdown	sin_Weekly $\times$ Lockdown
cos_Weekly	= $cos(\frac{2 \pi t}{T_w})$,$T_w=7$	cos_Weekly_Lockdown	sin_Weekly $\times$ Lockdown

According to $I_t = Y_t - S_t$ with the original time series $Y_t$, we can obtain the stationary series. Then, the lag order *p* of the stationary series $I_t$ was then determined using the PACF graph:

4 $$\begin{aligned} {\hat{I}}_t=f(I_{t-1}, I_{I-2}, \ldots , I_{t-p}). \end{aligned}$$

The optimal combination of distinct time lags is produced using the linear regression model *f*; ${\hat{I}}_t$ is the predicted value using the lag features of the sites. For each selected site, residual series are calculated as follows:

5 $$\begin{aligned} Z = I_t - {\hat{I}}_t. \end{aligned}$$

### Spatial autocorrelation variable

Spatial autocorrelation (SAC) reveals the similarity of the same feature between the target site and its neighbouring spatial sites^51^. Quantifying SAC avoids violating the assumptions underlying certain methods^52^, like machine learning, which dictates the independence of variables. Disobeying assumptions affects the performance of the model. In this study, we extract the SAC properties of the AQI from two perspectives, spatial dependence, and temporal autocorrelation. Statistically speaking, the temporal effect is one-dimensionally autocorrelated because the difference between any two time points is the same, regardless of the order between them. In contrast, the spatial effect is two-dimensional, and the degree is related to the Euclidean distance^53^. Thus, the SAC can be regarded as a two-dimensional extension of temporal autocorrelation with correlated degree inversely proportional to Euclidean distance between sites. In this paper, for the *i*-th site, we define its SAC variable as follows:

6 $$\begin{aligned} X_{SAC_i} = \sum ^n_{j = 1} \omega _{i,j} Z_j, \end{aligned}$$

where $\omega _{i,j}$ is the spatial weight between the *i*-th and *j*-th sites; *n* is the total amount of selected sites; and $Z_j$ is the residual series of the *j*-th site, calculated from Eq. (5). The weight $\omega _{i,j}$ is estimated with the kringing regression method considering the tuning spatial correlation function.

**Figure 3:**
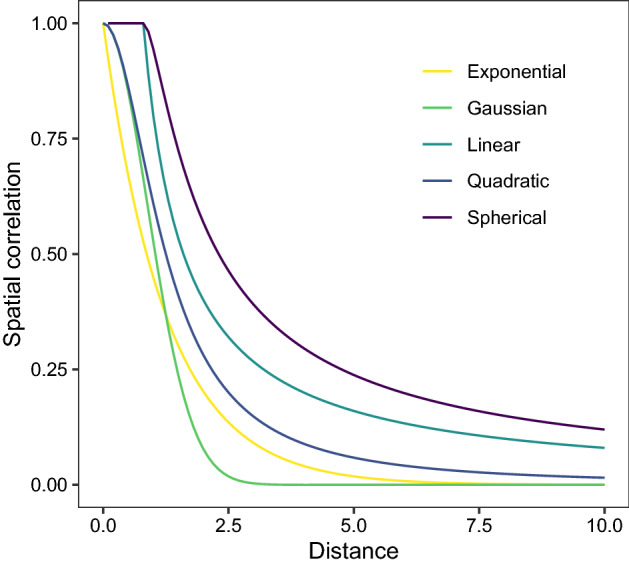
Variations in spatial correlation with distance when $\rho =0.8$.

In random fields, the spatial correlation between different locations of an attribute is represented by a spatially dependent correlation structure^54^. In this paper, we investigate five spatial correlation functions as follows:

- Exponential Correlation Function: $\alpha = e ^{- \rho d}$;
- Gaussian Correlation Function: $\alpha = e ^{- (\rho d)^2}$;
- Quadratic Correlation Function: $\alpha = \frac{1}{1+(\rho d)^2}$;
- Linear Correlation Function: $\alpha = 1- (1-\frac{\rho }{d} ) {\mathbb {I}}( \rho < d)$; and
- Spherical Correlation Function: $\alpha = 1-(1-1.5\frac{\rho }{d} + 0.5 (\frac{\rho }{d})^3){\mathbb {I}}( \rho < d)$,

where *d* denotes the Euclidean distance; $\rho$ is the parameter, and ${\mathbb {I}}$ is the characteristic function. Fig. 3 illustrates these five common spatially related structures^55^ where the trend of each spatially relevant structure is different in the same case of $\rho =0.8$, so selecting a suitable spatial correlation function is crucial for improving prediction accuracy.

#### The optimal SAC variable

The optimal SAC variable will be selected based on KNN-MI. The KNN-MI between the SAC variable and the dependent variable is calculated as follows:

7 $$\begin{aligned} I(X_{\text {SAC}} ; Y) \approx \psi (k)-<\psi \left( n_{X_{\text {SAC}}}+1\right) +\psi \left( n_{Y}+1\right) >+\psi (N). \end{aligned}$$

### Feature selection

In the QBSO (Q-learning based bee swarm optimization) algorithm, the solution vector $v={v_1,v_2, \ldots ,v_n}$ denotes the selected feature set, where $v_1=1$ means that feature $v_1$ is selected and 0 means it is discarded. There are multiple combinations of vector *v*, all of which form an *n*-dimensional state space *C*. We use KNN as the classifier, and the main process of the QBSO algorithm is as follows:

- Define an initial search feature solution $$\vartheta _0$$, and the solution is saved in a table named $$ {Solution}$$ to ensure that the solution is not repeatedly searched later.
- The search region (named $$ {SR}$$) of bees is determined by $$\vartheta _0$$, and the search region consists of multiple solutions. While searching, the bees exchange the obtained *Q* value with other bees and store it in the table $$ {Reward}$$, where the *Q* value is updated according to:
  8 $$\begin{aligned} Q\left( c, a\right) \leftarrow (1-\beta ) \cdot Q(c, a)+\beta \cdot \left( q +\gamma \cdot \max Q\left( c^{\prime }, a^{\prime }\right) \right) , \end{aligned}$$
  where $\beta \in [0,1]$ is a learning rate; and $\gamma$ is a discount parameter. When $\gamma \rightarrow 0$, the bee is more likely to choose the current reward, and if $\gamma \rightarrow 1$, the bee prefers to think about the future reward. The calculation of *q* is shown as follows:
  9 $$\begin{aligned} \left\{ \begin{array}{l} q_{t} \leftarrow {ACC }\left( h_{t+1}\right) \text{ if } {ACC }\left( h_{t}\right)< {ACC }\left( h_{t+1}\right) , \\ q_{t} \leftarrow {ACC }\left( h_{t+1}\right) - {ACC }\left( h_{t}\right) \text{ if } {ACC }\left( h_{t}\right)> {ACC }\left( h_{t+1}\right) , \\ q_{t} \leftarrow \frac{1}{2} \times {ACC }\left( h_{t+1}\right) \text{ if } {NUM } \left( h_{t}\right) > {NUM }\left( h_{t+1}\right) , \\ q_{t} \leftarrow -\frac{1}{2} \times {ACC }\left( h_{t+1}\right) \text{ if } {NUM}\left( h_{t}\right) < {NUM}\left( h_{t+1}\right) , \end{array}\right. \end{aligned}$$
  where $h_t$ denotes the current state; when the bee is at $h_t$, the set of actions that it may choose is $A_t = \left\{ a_{t_1} , a_{t_2} , \ldots , a_{t_n}\right\}$; ${NUM}\left( h_{t}\right)$ measures the amount of the feature subset at $h_t$; and ${ACC }\left( h_{t}\right)$ represents the classification accuracy based on the feature subset gained at $h_t$. In the QBSO algorithm, different classifiers can be selected, and the calculation of the classification accuracy *ACC* is as follows:
  10 $$\begin{aligned} {ACC } = \frac{\text {Amount of true positive }+\text {Amount of true negative }}{\text { Total amount of samples }}. \end{aligned}$$
  During this search, the bee chooses the solution $Ref_1$ maximizes *Q*.
- Repeat Step 2 until all $\vartheta _0$ have been obtained.
- Evaluate all $\vartheta _0$, using the classification accuracy of KNN as the first evaluation criterion and the feature set size as the second evaluation criterion, we can determine the optimal feature set.

### The forecasting model

In this work, LSTM and Bi-LSTM are used as the final predictors and both can be replaced. In addition, a feedforward neural network (FNN), RNN, and encoder-decoder LSTM (ENDC-LSTM) are chosen as benchmark models to illustrate the superiority of the target predictor. All these models are well suited to deal with time series problems. The following is a brief description of those benchmarks:

- FNN^56^: FNN is the most basic and classical form of neural network. It contains multiple hidden layers of neural networks, and the layers are fully connected to each other. The neurons are arranged in layers. Neurons only connect with neurons in the previous layer. The previous layer’s output is received and outputted to the next layer. Feedback between layers is not present.
- RNN^57^: In the traditional neural network, the layers are fully connected to each other, but the nodes between each layer are disconnected. This network is inefficient and unable to solve the dependency problem when dealing with sequences. RNN can solve this problem. In RNN, the current output of a sequence is related to the past output. This form allows the network to store the past information and apply it to the present output; briefly speaking, the input of the hidden layers contains the output of the input layer and the output of the hidden layer at the last time.
- ENDC-LSTM^58^: In practice, there are a large number of cases where the input and output sequences are of unequal length; some scholars design a network framework for mapping a variable-length sequence to another variable-length sequence, namely the encoder-decoder. This framework combined with LSTM can implement back-and-forth mapping between time sequences.

These network parameters are automatically adjusted using the *Optuna* package in Python.

**Table 2:** Candidate variables used to predict AQI.

Meteorological data		Air quality data	
Variable	Unit	Variable	Unit
Temperature	$^{\circ }$C	$\text {PM2.5}$	$\upmu {\text {g}}$ /${\text {m}}^3$
Humidity	%	$\text {PM10}$	$\upmu {\text {g}}$ /${\text {m}}^3$
Pressure	hpa	$\text {SO}_{2}$	$\upmu {\text {g}}$ /${\text {m}}^3$
Visibility	km	$\text {NO}_{2}$	$\upmu {\text {g}}$ /${\text {m}}^3$
Rainfall	mm	$\text {O}_{3}$	$\upmu {\text {g}}$ /${\text {m}}^3$
Cloudiness	%	$\text {CO}_{2}$	$\upmu {\text {g}}$ /${\text {m}}^3$
Wind speed	m/s		

## Case study

### Data collection

**Figure 4:**
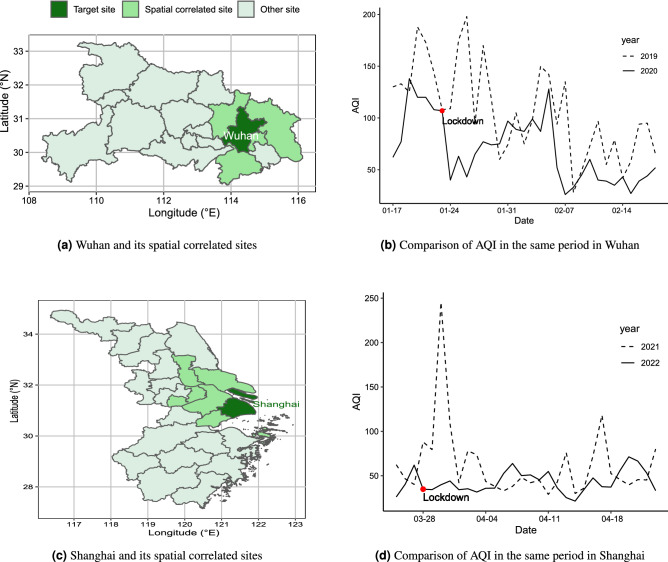
The study area and the variation of AQI.

Wuhan, the first city in China to be hit by COVID-19, implemented a lockdown policy to prevent the disease from spreading to other cites from January 23, 2020, to April 8, 2020. In 2022, the virus outbreak occurred again in Shanghai, and Shanghai has implemented city-wide containment management procedures since March 28, 2022. The lockdown policy refers to the static area management of the whole city, and residents are prohibited from going out to reduce the flow of people and cut off the transmission of the epidemic. To explore the impact of the lockdown policy on air quality, this paper selected data before and after the outbreak of COVID-19. The data from Wuhan cover the period from September 1, 2019, to December 31, 2020. At the time of our data collection, Shanghai was still under the lockdown, so the data for Shanghai were only retained until the day before the time of data collection (from January 1, 2021, to April 23, 2022). Fig. 4a,c are maps of Wuhan and Shanghai, and their surrounding cites. Figures 4b,d show the changes in AQI over a period after the start of the lockdown policy and a comparison of the AQI values at the same time in the past, with the red dots corresponding to the time points indicating when the lockdown policy was in place.

We collected daily data from 23 cites, including Shanghai, Wuhan, and their surrounding areas. The data of each city are composed of two parts (Table 2): (1) Air quality data come from the air quality platform (https://www.aqistudy.cn/), including AQI, $\text {PM2.5}$, $\text {PM10}$, $\text {SO}_{2}$, $\text {NO}_{2}$, $\text {O}_{3}$ and $\text {CO}_{2}$. (2) Meteorological data, including temperature, humidity, pressure, visibility, rainfall, cloudiness, and wind speed, come from the Huiju website (http://hz.hjhj-e.com/home/). A multiple interpolation from the MICE package in R is used to fill in the missing data of some of the meteorological variables in Wuhan. Initially, the air quality data for Shanghai were obtained on an hourly basis, so they were averaged to estimate the daily data. To eliminate the influence of measurement, we standardized all the data as follows:

11 $$\begin{aligned} x^{*}=\frac{x-\mu }{s_d}, \end{aligned}$$

where $\mu$ is the mean of *x*, and $s_d$ is the standard variance of *x*.

### Evaluation criterion

In this paper, we evaluate the performance of the model based on three metrics, including mean absolute error (MAE), mean absolute percentage error (MAPE), and root mean square error (RMSE). A model with smaller values is better. The following are the definitions of each index:

12 $$\begin{aligned}{} & {} \text {MAE} = \frac{1}{M} \sum _{i=1}^{M}\vert \left( y_{i}-\hat{y_{i}}\right) \vert , \end{aligned}$$

13 $$\begin{aligned}{} & {} \text {RMSE} = \sqrt{\frac{1}{M} \sum _{i=1}^{M}\left( y_{i}-\hat{y_{i}}\right) ^{2}}, \end{aligned}$$

and

14 $$\begin{aligned} \text {MAPE} = \frac{100 \%}{M} \sum _{i=1}^{M}\vert \frac{{y}_{i}-\hat{y_{i}}}{y_{i}}\vert , \end{aligned}$$

where *M* is the number of samples in the test set; *y* is the actual value, and ${\hat{y}}$ is the predicted value.

**Table 3:** The models compared and their definition.

Model	Abbreviation	Definition
Benchmark model	FNN	Feedforward neural network
	RNN	Recurrent neural network
	LSTM	Long short-term memory network
	Bi-LSTM	Bidirectional LSTM
	EN-DC LSTM	Encoder-decoder LSTM
	SAC-FNN	FNN with a spatial auto-correlation variable
	SAC-RNN	RNN with a SAC variable
	SAC-LSTM	LSTM with a SAC variable
	SAC-Bi-LSTM	Bi-LSTM with a SAC variable
	EN-DC LSTM	ENDC LSTM with a SAC variable
	QBSO-FNN	FNN with Q-Learning Based Bee Swarm Optimization
	QBSO-RNN	RNN with QBSO
	QBSO-LSTM	LSTM with QBSO
	QBSO-BiLSTM	Bi-LSTM with QBSO
	QBSO-ENDCLSTM	ENDC-LSTM with QBSO
	SAC-QBSO-FNN	FNN with QBSO adding a SAC variable
	SAC-QBSO-RNN	RNN with QBSO adding a SAC variable
	SAC-QBSO-ENDCLSTM	ENDC-LSTM with QBSO adding a SAC variable
Proposed Model	SAC-QBSO-LSTM	LSTM with QBSO adding a SAC variable
	SAC-QBSO-BiLSTM	Bi-LSTM with QBSO adding a SAC variable

### The experimental results

The four main objectives of the experiment in this study are to: (1) consider whether the lockdown policy will improve the forecasting accuracy; (2) confirm that the SAC variable selected by KNN-MI is optimal; (3) determine whether the QBSO algorithm improves the model performance, and (4) validate the effectiveness of the hybrid framework. We train some models to achieve these goals, and they are listed in Table 3. To avoid overfitting, the cross-validation method is adopted to divide the original data into training, validation, and test sets at an 8 : 1 : 1 ratio. The model is fitted on the training set. The validation set is used to tune the model parameters. After obtaining the optimal model through the training set and verification set, the test set is used to predict the model and evaluate the model performance. To ensure that the network has sufficient long-term memory input and does not increase the computational complexity, the time window chosen in the experiment is 30 and the prediction step size is 7.

**Figure 5:**
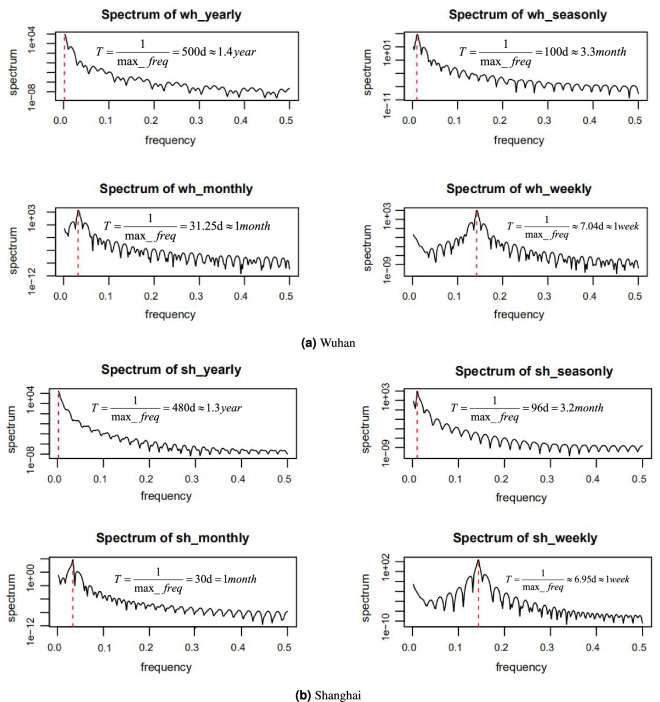
The spectrum of the yearly, seasonly, monthly, and weekly series of Wuhan and Shanghai.

#### Result of the COVID adjustment

From Fig. 4b,d, it can be seen that, after the lockdown policy was implemented, AQI dropped dramatically compared to the historical period. This is because traffic and factory pollution decreased during the lockdown. To this end, we need to eliminate the influence of these external factors. Only in this way can we better explore the potential laws of the data. Through trigonometric transformation, we abstract the yearly, seasonal, monthly, and weekly trends in the original series. Then we drew the spectrograms to verify periodic patterns in the decomposed series. In Fig. 5, the red asymptotes indicate the maximum frequency of each series, from which the period can be calculated. The spectrograms show that the yearly trend of the two cities is longer than 1 year, including 1.4 years for Wuhan and 1.3 years for Shanghai. This is because the amount of our data is limited. At least two years of data are needed to reflect the complete annual cycle. Despite that, AQI is empirically known to have that trend, so we still consider it to completely remove various trends from the original series. Furthermore, the other series show the corresponding distribution law.

**Figure 6:**
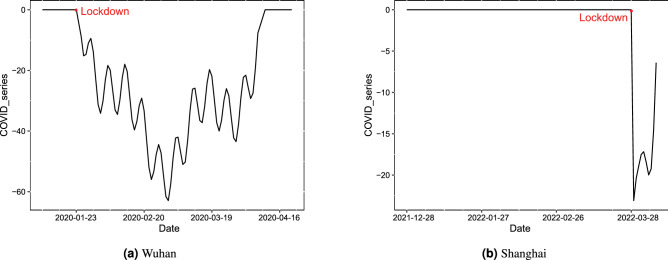
The impacts of the lockdown policy on AQI in two cities.

Since the data collected for Wuhan contain the complete lockdown period, it is mainly used here as an example to illustrate the effect of regression model adjustment (Fig. 6a). At the time of data collection in this paper, Shanghai had not yet ended its lockdown, so it is used as a secondary reference (Fig. 6b). To exclude the effects of trends and various cyclical patterns on the AQI series in the daily state, we have removed them. From Fig. 6, we can see that since the implementation of the lockdown policy, the series values have been negative; when not in lockdown, the values are 0. The lockdown policy has a generally negative effect on the AQI. This indicates that the lockdown policy will lower the AQI, which is consistent with the actual situation. Therefore, it is necessary to consider the impact of this policy when making forecasts.

**Figure 7:**
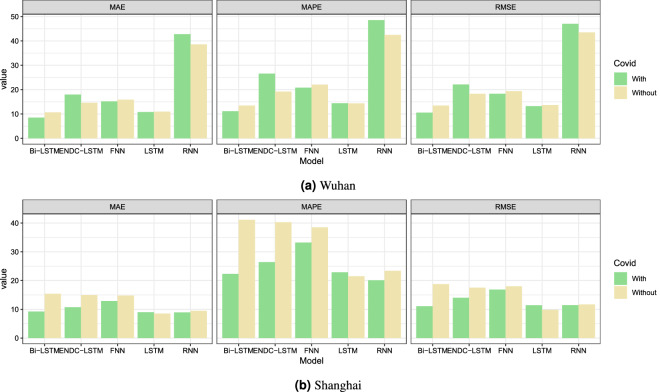
The impacts of COVID-19 on the different model for $t+1$ prediction.

To explore the impact of COVID-19 on forecasts, we then set up a control group without COVID-19 and an experimental group with it, and used all models to compare their predictive effects. Table 4 contains the 1-day, 3-day, and 7-day forecasts, and it can be observed that the prediction errors of most models decrease after considering the lockdown policy. The prediction accuracy improves significantly for Shanghai, in which the MAE of 1-day prediction of ENDC-LSTM drops from 14.97 to 10.74, a decrease of 28.2%, while 3-day and 7-day forecasts show decreases of 35.7% and 42.9%. For Wuhan, the first 3-day forecasts have a significant improvement. For example, the MAE for the 1-day forecast decreases from 10.68 to 8.52 by 20.2%; the RMSE and MAE decrease by 21.7% and 17.2%, respectively. For the 3-day forecast, the MAE, RMSE and MAPE decrease by 21.6%, 7% and 6.2%, respectively. In addition, we also visualize the results in Fig. 7. Taken together, the long-term forecasts for Wuhan are not convincing since there are many missing values in the original series. The sequence completed by the interpolation method cannot fully capture the real patterns of the data.

**Table 4:** The impacts of COVID-19 on the different model in Wuhan.

		Wuhan						Shanghai					
		COVID			Without-Covid			COVID			Without-Covid		
		t+1	t+3	t+7	t+1	t+3	t+7	t+1	t+3	t+7	t+1	t+3	t+7
FNN	RMSE	18.33	31.08	38.50	19.38	32.76	40.39	16.89	16.99	16.33	18.02	18.33	19.09
	MAE	15.19	22.51	29.33	15.91	23.77	30.33	12.89	12.72	11.83	14.78	15.13	15.61
	MAPE	20.8	28.48	36.24	22.09	29.72	36.57	33.24	32.64	29.35	38.53	39.23	39.11
RNN	RMSE	47.03	57.82	65.34	43.50	55.23	61.78	11.46	18.45	19.51	11.71	20.61	19.89
	MAE	42.78	46.87	51.89	38.58	43.73	48.43	8.91	14.21	15.55	9.49	17.07	16.64
	MAPE	48.57	46.67	51.43	42.49	43.11	47.28	20.10	32.07	33.6	23.40	44.24	40.46
LSTM	RMSE	13.24	31.04	37.50	13.69	30.72	38.72	11.42	17.04	16.86	9.84	22.28	20.35
	MAE	10.83	22.20	27.31	10.99	22.18	28.72	9.00	13.44	12.17	8.53	18.75	16.91
	MAPE	14.42	29.45	32.21	14.38	29.84	34.91	22.87	34.41	29.82	21.54	51.44	43.88
Bi-LSTM	RMSE	10.57	31.37	42.69	13.50	33.82	40.48	11.09	23.26	25.29	18.78	22.01	22.42
	MAE	8.52	23.22	30.30	10.68	24.92	29.60	9.22	18.55	19.07	15.42	18.46	18.72
	MAPE	11.16	30.94	33.34	13.48	32.98	36.23	22.33	50.05	47.21	41.15	49.29	48.05
ENDC-LSTM	RMSE	22.12	31.88	36.30	18.33	31.26	39.10	14.04	17.27	18.25	17.52	24.14	28.51
	MAE	17.99	23.45	27.81	14.63	22.33	28.17	10.74	13.24	13.61	14.97	20.59	23.82
	MAPE	26.59	31.33	36.76	19.22	28.48	34.99	26.44	33.88	33.21	40.31	56.60	61.82

#### The optimal SAC variable selected by KNN-MI

Before constructing the SAC variable, the spatially correlated sites corresponding to each target site need to be determined. After adjusting the original AQI series of each site, the Pearson correlation coefficients $\rho$ between the sites were calculated, and those with $\rho \ge 0.7$ were the spatially correlated sites. Table 5 contains the latitude, longitude and correlation coefficients of the target sites and their spatially correlated sites.

To find the best SAC variable, the KNN-MI statistic is utilized in this paper. Table 6 shows the KNN-MI between the AQI of the two target sites and the five SAC variables. The higher the value of KNN-MI is, the stronger the dependence between the two. The bolded values in the table are the best SAC variables for each site, and the best SAC variables are added to the hybrid framework proposed in this paper for prediction with other SAC variables. Tables 9 and 10 show the prediction results, and the SAC variables selected by the KNN-MI are indeed the ones that can improve the model performance the most.

**Table 5:** The latitude, longitude and $\rho$ of the target site and their spatially correlated sites.

	City	Longitude ($^{\circ }$E)	Latitude ($^{\circ }$N)	$\rho$
Wuhan	Wuhan	114.31	30.52	1.0000
	Xiaogan	113.91	31.92	0.8446
	Ezhou	114.88	30.40	0.8591
	Xianning	114.28	29.87	0.7706
	Huanggang	114.87	30.44	0.8610
Shanghai	Shanghai	121.48	31.22	1.0000
	Jiaxing	120.75	30.76	0.7339
	Nantong	121.05	32.08	0.8368
	Suzhou	120.62	31.30	0.7339
	Taizhou	119.92	32.48	0.7055
	Wuxi	120.29	31.59	0.7773
	Zhoushan	122.11	30.02	0.7066

#### Result of the QBSO

**Figure 8:**
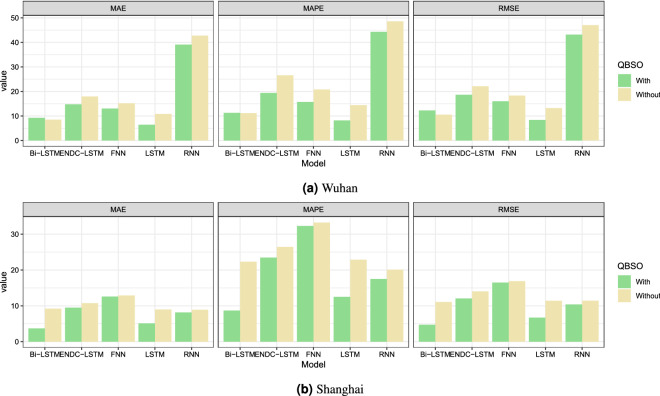
The impacts of QBSO on the different model for $t+1$ step prediction.

Feature selection is a common method to improve model performance. In this study, the QBSO algorithm parameters were manually tuned, with the learning rate $\lambda = 0.9$ and discount parameter $\gamma = 0.1$. Table 7 lists the number of original and filtered features that predict the AQI for each site, along with the classification accuracy and the average time to evaluate a solution. Table 7 shows that the QBSO algorithm can quickly determine whether a solution is correct and can achieve high accuracy. We employ the optimal set of features produced from the QBSO and the original feature set for prediction to ensure that it can truly improve model efficiency. In order to verify the effectiveness of the QBSO algorithm, we set up a control group without QBSO and an experimental group with it and conducted experiments using all models, and the experimental results were saved in Table 8. Figure 8 shows that the QBSO algorithm can improve the 1-day prediction accuracy of all the models effectively. To specific, for Wuhan, the 1-day prediction’s MAE of LSTM decreases from 10.83 to 6.45 dropped by 40.4%. For Shanghai, the 1-day forecast’s MAE of Bi-LSTM dropped by 60% from 9.22 to 3.69; the 3-day and 7-day declines were 31.8% and 40.7%, respectively. The QBSO algorithm significantly improves the performance of each model when predicting the AQI for Shanghai. The 3-day forecast and subsequent multistep predictions for Wuhan may fail to meet expectations because the original Wuhan dataset has many missing values, and the interpolated values cannot completely represent the real data.

**Table 6:** The KNN-MI between each SAC variable and dependent variable.

	Exponential	Gaussian	Quadratic	Spherical	Linear
Wuhan	**0.475**	0.451	0.400	0.445	0.383
Shanghai	0.557	**0.575**	0.544	0.547	0.370

Significant values are in bold.

**Table 7:** The performance of the QBSO on the different dataset.

	Wuhan	Shanghai
Amount of the original features	14	14
Amount of the selected features	4	6
Classification accuracy	91.22%	93.13%
Average time to evaluate a solution	0.017 s	0.032 s

**Table 8:** The results of QBSO on forecasting.

Wuhan								Shanghai					
		QBSO			Without-QBSO			QBSO			Without-QBSO		
		t+1	t+3	t+7	t+1	t+3	t+7	t+1	t+3	t+7	t+1	t+3	t+7
FNN	RMSE	16.02	34.08	38.42	18.33	31.08	38.5	16.50	16.46	16.08	16.89	16.99	16.33
	MAE	13.06	25.28	29.11	15.19	22.51	29.33	12.60	12.30	11.63	12.89	12.72	11.83
	MAPE	15.72	31.93	38.76	20.8	28.48	36.24	32.31	31.07	28.31	33.24	32.64	29.35
RNN	RMSE	43.16	60.59	62.38	47.03	57.82	65.34	10.40	16.76	16.58	11.46	18.45	19.51
	MAE	39.11	49.63	50.09	42.78	46.87	51.89	8.17	12.78	12.88	8.91	14.21	15.55
	MAPE	44.31	49.61	49.80	48.57	46.67	51.43	17.50	28.70	27.94	20.10	32.07	33.60
LSTM	RMSE	8.41	36.06	40.36	13.24	31.04	37.50	6.71	16.51	16.44	11.42	17.04	16.86
	MAE	6.45	26.34	29.32	10.83	22.20	27.31	5.13	12.39	11.89	9.00	13.44	12.17
	MAPE	8.20	32.06	36.43	14.42	29.45	32.21	12.51	30.93	28.90	22.87	34.41	29.82
Bi-LSTM	RMSE	12.25	35.58	40.29	10.57	31.37	42.69	4.73	16.91	17.97	11.09	23.26	25.29
	MAE	9.27	26.23	29.78	8.52	23.22	30.30	3.69	12.66	13.55	9.22	18.55	19.07
	MAPE	11.29	33.19	38.41	11.16	30.94	33.34	8.70	31.65	33.62	22.33	50.05	47.21
ENDC-LSTM	RMSE	18.67	34.27	40.83	22.12	31.88	36.30	12.05	16.48	17.67	14.04	17.27	18.25
	MAE	14.76	25.10	30.16	17.99	23.45	27.81	9.49	12.57	13.00	10.74	13.24	13.61
	MAPE	19.39	30.68	37.44	26.59	31.33	36.76	23.46	31.7	31.58	26.44	33.88	33.21

#### The comparison of the different predictors

**Figure 9:**
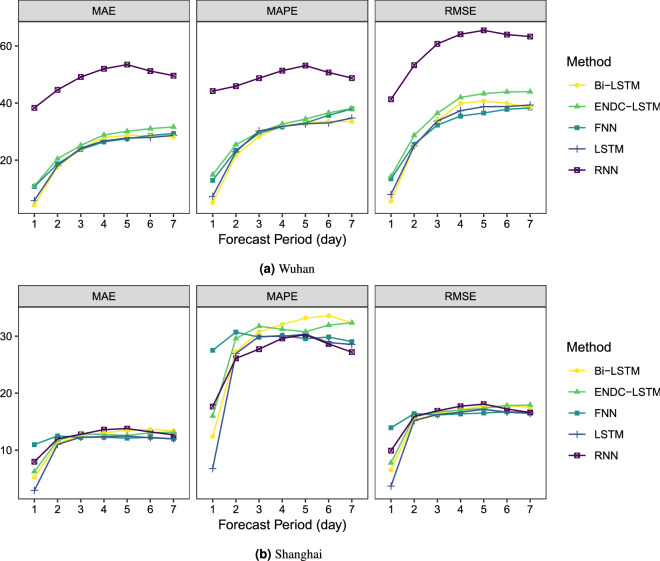
The results of the proposed model and other benchmarks.

**Figure 10:**
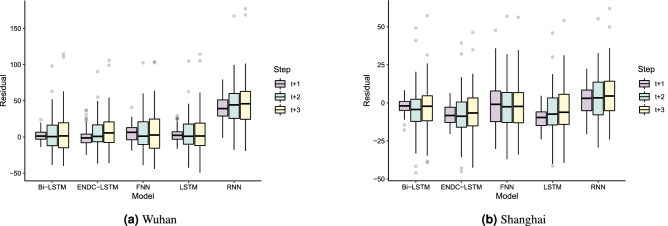
The residuals of the proposed model and other benchmarks.

In this subsection, we discuss the forecasting performance of the whole hybrid framework. From Table 6, it can be seen that the best SAC variable of Wuhan is exponential, while that of Shanghai is Gaussian. The corresponding best SAC variable is input into the framework to calculate the prediction accuracy, and the results are saved in Tables 9 and 10. Figure 9 compares the forecast errors over the next 7 days for Wuhan and Shanghai. RNN performs the poorest when predicting the AQI for Wuhan. This may be due to the poor fit of the interpolated missing values. In addition, RNN relies heavily on past values when predicting. The prediction error of the other networks rises significantly when the first three prediction steps are executed, and then stabilizes after 4 days. Combining the data in Table 9, the most suitable predictor for Wuhan is Bi-LSTM, whose 1-day forecast’s RMSE, MAE and MAPE were reduced by 47.2%, 49.6% and 54.2%, respectively; the 3-day error increased but not by much; the overall performance was better than the control group. For Shanghai, the accuracy of each network is close. Each model has a relatively low prediction error at 1-day and a relatively stable error change after the 2-day prediction. From Table 10, the most applicable predictor for Shanghai is LSTM, whose 1-day forecast’s RMSE, MAE, and MAPE are 3.68, 2.93, and 6.76, respectively, and the three evaluation indices are improved by 67.7%, 67.4% and 70.4%, respectively, compared with the control group. The performance of the proposed hybrid framework is excellent for datasets with complete information, such as Shanghai. From the residual box plot (Fig. 10), the LSTM and its extended form Bi-LSTM have error means that are closest to 0, as well as fewer outliers and modest error fluctuations; therefore, they can be utilized as predictors of the proposed framework in this research.

**Table 9:** The results of the proposed framework for Wuhan.

		Wuhan								
		Without			Exp-QBSO			Gau-QBSO		
		t+1	t+3	t+7	t+1	t+3	t+7	t+1	t+3	t+7
FNN	RMSE	18.33	31.08	38.5	13.46	32.26	38.26	17.15	32.02	38.33
	MAE	15.19	22.51	29.33	10.73	23.72	29.32	13.99	23.62	29.44
	MAPE	20.80	28.48	36.24	12.92	29.44	37.97	18.36	30.10	38.63
RNN	RMSE	47.03	57.82	65.34	41.32	60.73	63.29	46.10	59.66	61.87
	MAE	42.78	46.87	51.89	38.32	49.12	49.60	40.68	48.89	49.05
	MAPE	48.57	46.67	51.43	44.19	48.70	48.76	44.08	48.97	48.20
LSTM	RMSE	13.24	31.04	37.50	7.98	33.73	39.30	13.34	32.78	38.68
	MAE	10.83	22.2	27.31	5.91	24.19	28.66	10.36	23.78	28.78
	MAPE	14.42	29.45	32.21	7.31	30.22	34.74	14.25	31.13	37.79
Bi-LSTM	RMSE	10.57	31.37	42.69	**5.58**	**33.87**	**38.42**	19.36	32.59	38.66
	MAE	8.52	23.22	30.30	**4.29**	**23.91**	**28.18**	15.67	24.11	29.26
	MAPE	11.16	30.94	33.34	**5.11**	**28.33**	**33.53**	22.15	32.03	38.17
ENDC-LSTM	RMSE	22.12	31.88	36.30	14.31	36.43	43.96	19.83	32.44	36.64
	MAE	17.99	23.45	27.81	10.95	25.16	31.62	15.63	23.48	27.56
	MAPE	26.59	31.33	36.76	14.90	29.74	38.08	22.93	29.62	34.09
		Qua-QBSO			Lin-QBSO			Spher-QBSO		
		t+1	t+3	t+7	t+1	t+3	t+7	t+1	t+3	t+7
FNN	RMSE	16.95	31.71	38.29	15.45	31.65	38.40	15.84	31.78	38.48
	MAE	13.64	23.50	29.45	12.12	23.64	29.46	12.64	23.57	29.49
	MAPE	18.14	29.94	38.28	15.66	29.65	38.17	16.29	29.83	38.71
RNN	RMSE	46.59	60.02	61.23	41.30	59.90	63.78	49.05	60.86	62.44
	MAE	40.68	49.41	48.85	37.11	49.03	50.34	42.35	49.97	50.05
	MAPE	43.83	49.85	47.83	42.47	49.40	50.10	45.23	50.26	48.91
LSTM	RMSE	8.80	33.35	39.83	13.61	31.57	37.96	12.33	33.35	38.27
	MAE	6.78	23.94	29.5	10.41	22.92	28.96	9.35	24.27	29.01
	MAPE	8.59	30.03	36.81	14.09	29.97	38.11	12.60	30.84	37.66
Bi-LSTM	RMSE	9.89	37.09	40.72	11.53	31.67	37.55	14.64	30.51	38.50
	MAE	7.47	25.04	29.01	9.02	23.22	27.92	11.12	22.50	29.02
	MAPE	9.1	29.16	33.62	11.49	29.43	35.22	14.83	29.58	37.07
ENDC-LSTM	RMSE	18.73	32.58	36.67	13.17	32.39	41.04	18.01	31.51	37.16
	MAE	14.75	23.34	27.80	10.09	22.75	29.18	13.98	22.85	28.13
	MAPE	20.43	28.30	33.36	13.03	26.58	33.42	19.43	28.80	35.72

Significant values are in bold.

**Table 10:** The results of the proposed framework for Shanghai.

		Shanghai								
		Without			Exp-QBSO			Gau-QBSO		
		t+1	t+3	t+7	t+1	t+3	t+7	t+1	t+3	t+7
FNN	RMSE	16.89	16.99	16.33	13.48	16.44	16.56	13.93	16.18	16.67
	MAE	12.89	12.72	11.83	10.90	12.55	12.03	10.98	12.24	11.97
	MAPE	33.24	32.64	29.35	27.58	30.83	29.19	27.53	29.85	29.05
RNN	RMSE	11.46	18.45	19.51	10.54	16.73	16.67	9.91	16.90	16.60
	MAE	8.91	14.21	15.55	8.52	12.74	12.56	7.96	12.78	12.69
	MAPE	20.10	32.07	33.60	19.7	27.54	26.8	17.65	27.73	27.21
LSTM	RMSE	11.42	17.04	16.86	5.35	16.10	16.37	**3.68**	**16.24**	**16.45**
	MAE	9.00	13.44	12.17	4.24	12.22	11.82	**2.93**	**12.27**	**11.97**
	MAPE	22.87	34.41	29.82	10.10	29.16	28.74	**6.76**	**29.95**	**28.55**
Bi-LSTM	RMSE	11.09	23.26	25.29	11.32	17.04	17.00	6.52	16.40	17.52
	MAE	9.22	18.55	19.07	8.84	12.70	12.58	5.23	12.49	13.39
	MAPE	22.33	50.05	47.21	22.17	30.83	30.22	12.39	30.77	32.38
ENDC-LSTM	RMSE	14.04	17.27	18.25	14.44	16.60	17.16	7.77	16.73	17.92
	MAE	10.74	13.24	13.61	11.08	12.38	12.46	6.25	12.84	13.15
	MAPE	26.44	33.88	33.21	27.32	30.86	29.96	16.01	31.76	32.36
		Qua-QBSO			Lin-QBSO			Spher-QBSO		
		t+1	t+3	t+7	t+1	t+3	t+7	t+1	t+3	t+7
FNN	RMSE	13.23	16.31	16.34	15.65	16.09	15.81	15.27	16.52	16.81
	MAE	10.41	12.27	11.72	11.95	12.08	11.40	11.86	12.42	12.25
	MAPE	26.42	30.31	28.79	30.55	29.79	27.86	29.60	30.45	29.90
RNN	RMSE	10.14	16.95	16.78	9.68	17.25	17.82	8.34	16.96	17.03
	MAE	8.21	12.93	12.86	7.83	13.33	13.93	6.83	12.90	13.21
	MAPE	18.59	28.22	27.34	17.19	29.44	29.93	15.20	28.15	28.23
LSTM	RMSE	4.41	16.33	16.52	6.51	16.08	16.50	5.41	16.10	16.47
	MAE	3.63	12.37	12.25	5.08	12.19	11.97	4.38	12.23	12.20
	MAPE	8.65	29.83	28.89	12.33	29.43	28.66	10.52	29.86	28.47
Bi-LSTM	RMSE	4.48	16.82	17.99	7.40	16.84	17.41	11.98	16.40	16.49
	MAE	3.71	12.90	13.89	5.89	12.82	13.37	9.30	12.45	12.03
	MAPE	8.61	32.08	34.31	14.17	30.60	32.00	23.64	29.88	28.61
ENDC-LSTM	RMSE	12.08	16.39	17.55	12.23	16.50	17.26	13.23	16.62	17.91
	MAE	9.48	12.53	12.92	9.53	12.57	12.79	10.16	12.50	12.95
	MAPE	23.27	30.55	30.72	23.42	30.44	29.85	25.53	30.84	31.33

Significant values are in bold.

### General discussion

We find that the lockdown policy reduced traffic and factory pollution due to restricted human activities, and hence better air quality indexes. This confirms the findings of Tadano et al.^59^ and Al-qaness et al.^60^. Similarly, we find that LSTM and Bi-LSTM are robust tools for long-term AQI prediction, which is consistent with the findings of Xu and Yoneda^29^ and Zhang et al.^61^. There is a strong correlation between the AQIs of the target city and its neighboring cities, as well as historical data. It is different from the conclusion of Singh et al.^62^ that air quality can only be affected by pollutants and meteorological factors. Also, we confirm the importance of spatiotemporal pattern of AQI, emphasizing the need for joint pollution control at a regional level, which is in line with Tao et al.^63^.

## Conclusions

In this paper, we have proposed a deep learning framework for air quality prediction. Specifically, we have quantified the impact from the lockdown policy on air quality. While analyzing the data, we have found that the AQI of the target city is highly correlated with some of its neighboring cities. For example, the AQI correlation coefficient between Wuhan and Xiaogan reaches 0.86 while that between Shanghai and Nantong is 0.84. More generally, this provides a new idea for predicting AQI, that is, to consider the impact brought by AQI of spatially related cities. The experimental results prove that this approach is feasible. Furthermore, in our proposed framework, we have found the LSTM and Bi-LSTM among all considered baseline algorithms can provide highly accurate long-term predictions for our two cases.

Some other directions can be further explored for improving AQI forecasting. First, the severity of the lockdown restrictions often varies from time to time, thus, to obtain a more accurate evaluation, we can distinguish the different lockdown policies and investigate their impact on AQI. Second, the air quality may be affected by many other factors including fuel prices, public holidays and other environmental protection policies. Incorporation of these factors should also improve the forecast. Thirdly, our work focuses on air quality prediction for specific cities (e.g., Wuhan and Shanghai), so we are unable to simulate the spatial heterogeneity for individual cities. When there are many available air quality monitoring stations in a city, it is necessary to consider its spatial heterogeneity. In addition, an alternative spatial correlation modeling to our statistical approach, the graph network, can also be investigated for air quality forecasting performance. Last, although the QBSO algorithm is efficient for feature selection, according to our numerical results, the optimized performance for our proposed framework is dependent on the subjective selection of kernel functions, e.g., spatial correlation functions. Further work on development of selection criteria instead of cross validation for computation efficiency will be very valuable.

## Data Availability

The datasets generated and analysed during the current study are available via https://github.com/Zixizhao0/AQI-LSTM-prediction.

## Code availability

*Accession codes* A demo of the proposed method in this paper can be obtained by sending a request to the author (20461026005@ stu.wzu.edu.cn).
